# The Aphrodisiac and Androgenic Effects of Aqueous and Ethanol Extracts of *Tribulus cistoides* (Zygophyllaceae) on Nicotine-Induced Sexual Dysfunction in Male Wistar Rats

**DOI:** 10.1155/tswj/7410030

**Published:** 2025-10-17

**Authors:** Kiafon Betrand Nsah, Njoya Moses Tita Mogho, Nguedem Sylvie Fonkwo, Kada Sanda Antoine

**Affiliations:** Department of Zoology, University of Bamenda, Bambili, Cameroon

**Keywords:** androgenic, aphrodisiac, FSH, LH, male sexual dysfunction, testosterone, *Tribulus cistoides*

## Abstract

Male sexual dysfunction is a common condition, typically managed with phosphodiesterase Type 5 inhibitors and testosterone replacement therapy. However, these treatments often have undesirable side effects and are expensive, prompting interest in plant-based alternatives. This study investigated the aphrodisiac and androgenic potential of *Tribulus cistoides* (*T. cistoides*) in a nicotine-induced model of sexual dysfunction in male Wistar rats. A total of 45 male rats were divided into nine groups (*n* = 5 per group). Group 1 served as the normal control; Group 2 received nicotine only (1 mg/kg) to induce dysfunction; Group 3 received sildenafil citrate (5 mg/kg) as a positive control. Groups 4–6 were administered nicotine plus aqueous extracts of *T. cistoides* at doses of 50, 100, and 150 mg/kg, respectively. Groups 7–9 received nicotine plus ethanol extracts at the same doses. Sexual behavior was assessed on Days 1, 5, and 9, and animals were sacrificed on Day 10 for biochemical and histological analyses. Outcomes measured included reproductive organ weights, serum levels of testosterone, FSH, LH, oxidative stress markers, testicular protein content, and seminal vesicle fructose levels. Nicotine exposure impaired sexual behavior, as evidenced by increased mount latency and decreased mount frequency, intromission frequency, and penile licking. However, treatment with the plant extract effectively reversed these effects, restoring the sexual behavior parameters toward normal levels. Nicotine also significantly reduced testosterone levels (0.079 ± 0.006 ng/mL), while *T. cistoides* extracts markedly restored hormone levels to 1.002 ± 0.165 ng/mL (aqueous) and 0.865 ± 0.041 ng/mL (ethanol) and improved nitric oxide levels, and gonadotropin concentrations. Additionally, the extracts restored testicular oxidative balance and enhanced reproductive tissue biochemistry. These effects are likely mediated by bioactive phytochemicals such as saponins, alkaloids, and flavonoids, which may promote vasodilation, modulate hormone levels, and enhance nitric oxide production. Findings suggest that *T. cistoides* may offer a natural alternative for managing male sexual dysfunction with fewer side effects than conventional drugs.

## 1. Introduction

Male sexual dysfunction is characterized by erectile dysfunction, Peyronie's disease, premature ejaculation, low libido, and anorgasmia [1, 2]. Erectile dysfunction and premature ejaculation are the most common sexual dysfunctions in men [2, 3]. Studies have revealed that male sexual dysfunction has a direct relationship with age, with an estimated prevalence of 50% in men within the age range of 40–70 years old [4]. Erectile dysfunction has several causes, among which drugs like selective serotonin reuptake inhibitors, antihormonal drugs, recreational drugs, lifestyle habits, and health complications are important risk factors for sexual dysfunction [5]. Diagnoses of sexual dysfunction are challenging based on the fact that it is not life-threatening and symptoms are seldom reported. The first line of treatment involves oral phosphodiesterase Type 5 (PDE5) inhibitors [4]. Moreover, in combination with nitrates, it can cause fatal hypotension [6]. Hence, there is a need for alternative ways to manage sexual dysfunction in general and erectile dysfunction in particular.

Globally, an estimated one-third of men are active smokers, with a majority of them unaware that cigarette smoking can contribute to erectile dysfunction [7]. Other studies hold that the prevalence of tobacco consumption is estimated at 19% in the adult population, 33% among males, with nicotine being its major component and a risk factor of sexual dysfunction, which has been reported to have a dose-dependent effect on erectile dysfunction [8, 9]. Nicotine alters vasoreactivity through endothelium-dependent and/or endothelium-independent mechanisms, hence hindering penile erection. Its ability to disrupt androgen and nitric oxide (NO) production makes nicotine a potential inducer of sexual dysfunction. According to research, nicotine damages nerves involved in sexual arousal and also causes depression and anxiety in individuals addicted to cigarette smoking, which are causes of male sexual dysfunction [10–12].

Medicinal plants contain bioactive molecules that can serve as alternatives to pharmaceutical treatments and as sources of new biomolecules for the management of male sexual dysfunction [13]. Therefore, this study was aimed at determining the aphrodisiac properties of ethanol and aqueous extracts of *Tribulus cistoides* on nicotine-induced sexual dysfunction in male albino rats (Wistar rats). *The T. cistoides* plant is used in traditional medicine in several African countries, including Cameroon, where it is used in the treatment of male sexual dysfunction. In Asia, *T. cistoides* is used traditionally for the treatment of malaria, for infections of the kidney and bladder, as a cure for toothache, as an aphrodisiac, and for the treatment of male infertility [14–16]. There is no scientific evidence supporting the use of *T*. *cistoides* in the management of male sexual dysfunction. Hence, this study was aimed at evaluating the aphrodisiac properties of the ethanol and aqueous extract of *T. cistoides* on nicotine-induced sexual dysfunction in male albino Wistar rats.

## 2. Materials and Methods

### 2.1. Plant Collection and Powder Preparation

The entire *T. cistoides* plant was collected in February 2024 from Kousseri, located in the Far North Region of Cameroon, at geographic coordinates approximately 12.0762° N latitude and 15.0308° E longitude, and an elevation ranging from 270 to 283 m above sea level. The plant was identified at the Cameroon national herbarium in comparison with the materials of the collector Moussa 01 of the specimen in the herbarium Collection Number 67586/HNC of the Cameroon national herbarium. The harvested plant material was washed using tap water and air-dried at ambient temperature for a period of 1 week and ground using an electric blender (Binatone model) and sieved to obtain the fine powder.

### 2.2. Extract Preparation

Aqueous and ethanol extracts of *T. cistoides* were prepared to capture a broad spectrum of phytochemicals with potential androgenic and aphrodisiac activity. Aqueous extraction was used to simulate traditional medicinal preparations and is known to extract primarily polar compounds such as saponins, flavonoids, and glycosides. Ethanol, being a semipolar solvent, was employed to extract a wider range of bioactive constituents, including both polar and nonpolar compounds such as alkaloids, steroidal saponins, and lipids. This dual-extraction approach was intended to maximize the representation of phytochemicals potentially involved in the observed biological activities.

#### 2.2.1. Preparation of Aqueous Extract

The aqueous extract of *T. cistoides* was prepared by decoction according to [17] with slight modification. Briefly, 200 g of the *T. cistoides* powder was boiled in 2 L of distilled water for 20 min and allowed to settle at room temperature. The preparation was filtered using Whatman Number 1 filter paper. The filtrate was evaporated using a thermostat oven (Model: DHG-9101-1 SA) at a temperature of 40°C to obtain the aqueous extract. The extract was weighed using an electronic balance (Model: JJ500). The mass of the aqueous extract obtained was 33.06 g, giving a 13.23% yield, which was scraped, transferred into a tightly labeled container, and stored in a refrigerator to be used in the experiment.

#### 2.2.2. Preparation of Ethanol Extract

The ethanol extract of *T. cistoides* was prepared by maceration according to the method of [18] with slight modification. Briefly, 200 g of the powder was soaked in 1 L of ethanol, incubated for 72 h, and filtered through Whatman filter paper Number 1. The filtrate was evaporated using a Thermostat Oven (Model: DHG-9101-1 SA) to dryness and weighed using an electronic balance (Model: JJ500). 9.03 g of the extract was obtained with a percentage yield of 4.51%.

### 2.3. Experimental Animals and Ethical Statement

A total of 45 male albino Wistar rats of 4 months old and 10 female albino Wistar rats (*Rattus norvegicus*), weighing between 150 and 200 g, were purchased from a breeder in the city of Bamenda Northwest Region of Cameroon. Animals were transferred to the animal house of the Department of Zoology, Faculty of Science of the University of Bamenda, Cameroon, where they were acclimatized for 2 weeks before experimentation. Animals were handled following the ethical guidelines of the Cameroon National Veterinary Laboratory guide as a reference approved for health control (No: 003/19 CCS/MINEPIA/RD-NW/DDME/SSV) from the start to the end of the experiment. The rats were given water and food ad libitum and were subjected to a 12:12-h light:dark cycle under standard laboratory conditions at a temperature of 24°C–28°C with a relative humidity of 60%–70%.

### 2.4. Induction of Estrus to Ovariectomized Female Rats for Sexual Behavior Tests

Estrus was induced on ovariectomized female rats by a sequential subcutaneous injection of estradiol benzoate (10 *μ*g/100 g of body weight) manufactured by Hebei New Century Pharmaceutical Co. Ltd., Address No: 159, Taihang street Shijiazhuang, Hebei, China, and Progesterone Retard Pharlon (500 *μ*g/100 g of body weight) manufactured by Bayer AG, 13342 Berlin, Germany, 48 and 5 h, respectively, before the testing time [19].

### 2.5. Animal Grouping and Treatments

Forty-five male Wistar rats were shared equally into nine groups of five rats each and were treated orally for 9 days as follows: Group 1 received distilled water 5 mL/kg (normal control); Group 2 received nicotine only 1 mg/kg (negative control); Group 3 received nicotine 1 mg/kg and Viagra 5.625 mg/kg (positive control); Groups 4, 5, and 6 received nicotine 1 mg/kg **+** *T. cistoides* aqueous extract at doses of 50, 100, and 150 mg/kg, respectively; Groups 7, 8, and 9 received nicotine 1 mg/kg + *T. cistoides* ethanol extract at the doses of 50, 100, and 150 mg/kg, respectively. The treatment duration was guided by the study of [20] on the acute evaluation of the aphrodisiac properties of plant extracts; the dose of nicotine was chosen based on the study of [12]. The dose of sildenafil citrate (SC), also called Viagra, was chosen according to [21], and *T*. *cistoides* extract doses were chosen according to [22]. On the 9th day, rats were fasted overnight and sacrificed on the 10th day.

### 2.6. Evaluation of the Effect of *T. cistoides* Extracts on Sexual Behavior Parameters

Using a standard cage (a round netted bowl with a height of 23 cm and a circumference of 707.14 cm^2^), the evaluation of the effect of the aqueous and ethanol extracts of *T. cistoides* on male copulatory behavior was done using male rats in the presence of receptive (estrus) female rats. During the 9 days' treatment period, male copulatory behaviors were tested on Days 1, 5, and 9 [20]. The following sexual behavior parameters were evaluated according to the method of [22, 23] with slight modification:

• *Mount latency*: Duration (in seconds) from the introduction of the female into the cage till the first mount.
• *Ejaculation latency*: Duration (in seconds) from the first intromission till the first ejaculation.
• *Mount frequency* (*MF*): Total number of mounts from the introduction of the female to the end of 15 min.
• *Intromission frequency* (*IF*): Total number of intromissions within 15 min of observation.
• *Ejaculatory frequency* (*EF*): The number of ejaculations within 15 minutes of observation.
• *Postejaculatory latency*: Duration (in seconds) from the first ejaculation to the next series of mounts.
• *Penile leaking frequency* (*PLF*): Total number of penile lickings within 15 min of observation.

Male rats were placed in individual cages for 5 min to acclimatize, estrous females were introduced into the cages, and the aforementioned sexual parameters were recorded during a 15-min observation.

### 2.7. Sacrifice, Collection of Blood Samples, and Reproductive Organs

On the 9th day of treatment, animals were fasted overnight and sacrificed on the 10th day. On the day of sacrifice, animals were anesthetized by intraperitoneal injection of the combination of diazepam (10 mg/kg) and ketamine (0.5 mg/kg). Thereafter, rats were sacrificed by cervical decapitation, and the venous blood samples were collected from the jugular veins of each rat into dry tubes. Tubes were centrifuged for 15 min (3000 revolutions per minute) to obtain serum. The serum collected was used for the quantification of luteinizing hormone (LH), follicle-stimulating hormone (FSH), and testosterone levels.

Animals were appropriately dissected to collect the reproductive organs: testes, epididymides, seminal vesicles, ventral prostates, and penis. Adipose tissues were carefully removed, and organs weighed using an electronic scale balance (G&G Electronic Scale: Model: JJ500). The left testis was collected and preserved in 10% formalin for histological examination. Organ weight index was calculated according to a method used by [24]:

 $$\begin{array}{c} \text{Organ index}=\left(\frac{\text{WO}}{\text{BW}}\right)\times 100 \end{array}$$

where WO is the organ weight and BW is the body weight.

### 2.8. Preparation of Testicular, Penile, and Seminal Vesicle Homogenates

The right testis, penis, and seminal vesicles were crushed in separate mortars using 20% phosphate buffer 0.1 M pH 7.2. The homogenized solutions were centrifuged using a Hettich EBA 3S centrifuge at 3000 rpm for 15 min, and the supernatants were used for biochemical analyses.

### 2.9. Biochemical Analysis

#### 2.9.1. Assessment of Testosterone, Luteinizing, FSH, and Oxidative Stress Biomarkers

The level of testosterone, luteinizing, and FSH was assessed using ELISA kits (Elabscience, Houston, Texas, United States) according to the manufacturer's procedure.

#### 2.9.2. Assessment Oxidative Stress Biomarkers

The concentration of glutathione (GSH), thiobarbituric acid reactive substance (TBARS), and catalase (CAT) activity was determined using the method described by [25]. NO was evaluated using the Griess assay [26], and superoxide dismutase (SOD) activity was determined as described earlier [27]. The concentration of testicular proteins and vesicular fructose was quantified using the method described by [28, 29], respectively.

### 2.10. Histopathological Examination of the Testis

The histopathological examination of the testis was done according to the method described by [24]. Shortly after sacrifice, the left testis was fixed with 10% neutral buffered formalin in separate containers. Samples were dehydrated using five grades of ethanol progressively (70%, 80%, 90%, 95%, and 100%). Ethanol was cleared by using two changes of xylene for 24 h, respectively, after which they were hardened by incubating for 24 h in two changes of molten paraffin wax and then embedded and blocked out. Sections of 5 *μ*m thickness were cut using a rotary microtome and mounted on glass slides using collagen solution (500 mg of collagen in 1000 mL of distilled water). Slides were carefully passed through three changes of xylene and changes of ethanol for 10 min each to remove paraffin and rinsed in distilled water for 5 min. The slides were stained with hematoxylin and eosin (H&E) according to [30]. Pictures of the stained sections of control and treated rats were captured under a light microscope (Olympus BX51) at 100× magnification lens.

### 2.11. Data Analysis

The data collected were analyzed using GraphPad Prism Version 9, and the results were presented as mean ± standard error of means (SEM) for each group. The differences among the respective groups were compared using one-way analysis of variance (ANOVA), and the difference between the means of the various treatments was compared using Tukey's multiple comparison test with statistical significance set at *p* ≤ 0.05.

## 3. Results

### 3.1. Effects of Aqueous and Ethanol Extracts of *T. cistoides* on Sexual Behavior of Male Rats Exposed to Nicotine

#### 3.1.1. Effect of Aqueous and Ethanol Extracts of *T. cistoides* on the Mount Latency (Seconds) of Male Rats Exposed to Nicotine

Treatment of male rats with nicotine (1 mg/kg of body weight) significantly increased (*p* ≤ 0.001) the mount latency compared to the normal group treated with distilled water (5 mL/kg of body weight). Treatment with the aqueous and ethanol extracts of *T. cistoides* significantly (*p* ≤ 0.001) reduced the mount latency compared to the nicotine-treated group in a dose-independent manner from the 1st day of treatment to the 9th day, as presented in Table 1. After comparing the effect of the two extracts using a *t*-test, from Days 1 to 9, there was no significant difference in the activity of the extracts except for the results on Day 5 of the treatment, which revealed that 50 mg/kg of ethanol extract (2.600 ± 0.509 s) of *T. cistoides* was better than that of the aqueous extract (10.80 ± 0.916 s).

#### 3.1.2. Effect of Aqueous and Ethanol Extracts of *T. cistoides* on the MF of Male Rats Exposed to Nicotine

As detailed in Table 2, treatment with nicotine on Day 1 produced no statistical difference in MF as compared to the normal control treated with distilled water. After evaluating the MF on Days 5 and 9, results showed that nicotine had significantly (*p* ≤ 0.01) reduced the MF as compared to the normal group treated with distilled water. It is interesting, as treatments with the aqueous and ethanol extracts on Day 5 significantly (*p* ≤ 0.001 and *p* ≤ 0.01) increased the MF in a dose-independent manner, respectively. On the 9th day of treatment, 50 and 100 mg/kg produced similar results to those on Day 5. One hundred fifty milligrams per kilogram of the plant extracts demonstrated a much greater significant (*p* ≤ 0.001) increase when compared to the nicotine-treated groups. Treatment with SC 5 mg/kg of body weight significantly (*p* ≤ 0.001) increased the MF of the positive control group. Generally, treatment of rats with *T. cistoides* ethanol and aqueous extracts induced an increase in MF from Days 1 to 9, while there was no statistical difference in the action of the extracts according to the results of the *t*-test.

#### 3.1.3. Effect of Aqueous and Ethanol Extracts of *T. cistoides* on Ejaculatory Latency (in Seconds) of Male Rats Exposed to Nicotine

Table 3 represents the effect of *T. cistoides* extracts on the ejaculatory latency of male rats exposed to nicotine. Nicotine significantly decreased the ejaculatory latency on Day 1 (*p* ≤ 0.001), Day 5 (*p* ≤ 0.05), and Day 9 (*p* ≤ 0.001) when compared to the normal control treated with distilled water. After analyzing the ejaculatory latencies of the extract-treated groups, it was noticed that the extracts significantly increased (*p* ≤ 0.001) the ejaculatory latencies on Days 1, 5, and 9, especially in the groups treated with 150 mg/kg of both extracts compared to the negative control group. Treatment with SC significantly increased the ejaculatory latency in the positive control group on Day 1 (*p* ≤ 0.001), and Day 5 (*p* ≤ 0.05), only when compared to the negative control group. The *t*-test results indicated that the actions of ethanol and aqueous extract were not statistically different.

#### 3.1.4. Results of the Effect of Aqueous and Ethanol Extracts of *T. cistoides* on EF of Male Rats Exposed to Nicotine

Treatment with nicotine showed a significant decrease (*p* ≤ 0.05 and *p* ≤ 0.01, respectively) in the EF as compared to the normal control treated with distilled water on Days 5 and 9. Treatments with ethanol and aqueous extracts on Day 1 increased the EF by 22% following treatment with 150 mg/kg of both extracts compared to the nicotine-treated group. On Day 5, treatment with 50 mg/kg of body weight of both extracts produced a 12% increase in EF when compared to the nicotine-treated group. One hundred and 150 mg/kg of ethanol extract significantly increased the EF (*p* ≤ 0.01 and *p* ≤ 0.001), while only 150 mg/kg of the aqueous extract produced a significant (*p* ≤ 0.01) increase in the EF. On Day 9 of the experiment, both extracts demonstrated a dose-dependent increase in EF as compared to the negative control, with 150 mg/kg indicating a much higher significant increase (*p* ≤ 0.001) for both extracts as tabulated in Table 4. The effect of both extracts was compared using a *t*-test, and results revealed that there was no significant difference (*p* ≥ 0.05) in the action of the extracts regarding their effect on EF.

#### 3.1.5. Effect of Aqueous and Ethanol Extracts of *T. cistoides* on Postejaculatory Interval

After recording and analyzing the postejaculatory latency of rats treated with nicotine, the postejaculatory interval of the normal control was higher than that of the negative by 14% on Day 1. Day 5 (*p* ≤ 0.05) and Day 9 (*p* ≤ 0.01) of the treatment witnessed respective increases in the postejaculatory intervals of the nicotine-treated groups as compared to the normal control, as shown in Table 5. Treatment with the plant extracts on Day 1 showed an insignificant decrease in postejaculatory interval with both extracts, with a 12.00% and 7.47% decrease for aqueous and ethanol extracts at 150 mg/kg of both extracts, respectively, when compared to the negative control. On Day 5 of the treatment, the postejaculatory latency was evaluated, and all the doses for the aqueous and ethanol extract significantly (*p* ≤ 0.05) decreased the postejaculatory interval compared to the negative control. In addition, 150 mg/kg of body weight of aqueous extract showed a greater significance (*p* ≤ 0.01) among the doses tested as compared to the negative control. On Day 9, treatment with the aqueous extract proved a dose-dependent effect evident in the group treated with 150 mg/kg, showing a much more significant (*p* ≤ 0.001) decrease as compared to the 50 and 100 mg/kg of the aqueous extract as compared to the negative control. Treatment with 100 mg/kg of body weight of ethanol extract showed a significant decrease with *p* ≤ 0.001, which was the same with 150 mg/kg of ethanol extract as compared to the negative control. The *t*-test results revealed no statistical difference in the action of both extracts.

#### 3.1.6. Effect of Aqueous and Ethanol Extracts of *T. cistoides* on PLF of Male Rats Exposed to Nicotine

Table 6 summarizes the effect of aqueous and ethanol extracts of *T. cistoides* on the PLF of nicotine-exposed male rats. Results revealed that exposing male rats to nicotine had a negative impact on the rats' sexual performance, manifested through the significant decrease in PLF on Day 1 (*p* ≤ 0.05), Day 5 (*p* ≤ 0.05), and Day 9 (*p* ≤ 0.001) as compared to the normal control treated with distilled water. Interestingly, treatment with both extracts corrected the decreased penile leaking impairment by significantly increasing the PLF as compared to the nicotine-treated group in a dose-dependent manner on Days 1 and 5. On Day 9, the effect of the ethanol extract was dose independent, where different doses produced the same level of significant increase (*p* ≤ 0.001) in PLF compared to the nicotine-treated group, while the aqueous extract maintained a dose-dependent effect. Following a comparison of the action of ethanol and aqueous extracts, *t*-test values showed that there was no statistical difference in the influence of the extract on PLF.

#### 3.1.7. Effect of Aqueous and Ethanol Extracts of *T. Cistoides* on IF of Male Rats Exposed to Nicotine

According to our results presented in Table 7, rats treated with nicotine showed a significant decrease in penile IF on Day 1 (*p* ≤ 0.001), Day 5 (*p* ≤ 0.05), and Day 9 (*p* ≤ 0.01) as compared to the normal control treated with distilled water. Most importantly, treatment with ethanol and aqueous extracts alleviated the decrease in IF induced by nicotine. On Day 1, treatment with 100 and 150 mg/kg of body weight of both extracts significantly increased the IF (*p* ≤ 0.001) more than 50 mg/kg (*p* ≤ 0.001) as compared to the nicotine-treated group. On Day 5 of the experiment, the aqueous extract demonstrated a dose-independent increase in IF, unlike the ethanol extract, with 100 and 150 mg/kg showing the same level of significant increase (*p* ≤ 0.001) as compared to the negative control treated with nicotine. On Day 9, the ethanol extract was confirmed to have increased IF in a dose-independent manner, unlike the aqueous extract, where 100 and 150 mg/kg significantly (*p* ≤ 0.001) increased IF more than 50 mg/kg (*p* ≤ 0.001) as compared to the negative control. Treatment with the reference drug, SC (5 mg/kg of body weight), maintained a significant (*p* ≤ 0.001) increase in IF compared to the negative control. Generally, after a comparison of the results of the two extracts using a *t*-test, there was no significant difference in their effect on the IF of male rats exposed to nicotine.

### 3.2. The Effect of *T. cistoides* Extracts on the Weights (Grams) of Reproductive Organs of Rats Exposed to Nicotine

In Table 8, treatment of animals with nicotine for a period of 9 days showed an insignificant decrease in the weight of all reproductive organs evaluated after comparing with the normal control treated with distilled water. Cotreatment with either the aqueous or the ethanol extract alongside nicotine at various doses resulted in an increase in the weight of reproductive organs compared to the negative control. After comparing the effect of distilled water on reproductive organ weight to that of nicotine, distilled water was found to have maintained an increase in the weight of some reproductive organs (epididymis [10%], seminal vesicle [6.7%], and penis [10%]). The testis and prostate did not show any increase in the normal control as compared to the negative control. After comparing the effect of the extracts on reproductive organ weight, treatment with 150 mg/kg of both extracts increased reproductive organs as follows: epididymis (aqueous extract 2.4% and ethanol extract 13.4%), testis (aqueous extract 0.001% and ethanol extract 8%), seminal vesicle (aqueous extract 17% and ethanol extract 15.5%), prostate (aqueous extract 25% and ethanol extract 25%), and penis (aqueous extract 18% and ethanol extract 18%). Similarly, treatment with SC 5 mg/kg of body weight demonstrated an insignificant increase in reproductive organs; likewise, a *t*-test conducted to experiment with the difference in the actions of both extracts showed no significant difference.

#### 3.2.1. Effect of Aqueous and Ethanol Extracts of *T. cistoides* on Testis Oxidative Stress Biomarkers

The oxidative stress biomarkers of the testicular homogenate were determined, and the results are presented in Table 9. The activity of the SOD was reduced significantly (*p* ≤ 0.001) by nicotine in the testis as compared to the normal group treated with distilled water. The activity of SOD was significantly increased by the ethanol and aqueous extracts in a dose-dependent manner as compared to the negative control.

Testicular levels of TBARS were found to be significantly higher (*p* ≤ 0.05) in the nicotine-treated group when compared to the normal control group. Fifty milligrams per kilogram of the aqueous extract normalized the concentration of TBARS by causing a significant decrease (*p* ≤ 0.05) compared to the negative control, while the same dose of ethanol extract did not show any significant decrease in the level of TBARS as compared to the negative control. One hundred and 150 mg/kg of the aqueous extract significantly (*p* ≤ 0.01) decreased the concentration of TBARS. The same doses of ethanol extract significantly (*p* ≤ 0.05) decreased the concentration of TBARS, while SC at 5 mg/kg of body weight could not restore the levels of TBARS.

Following the evaluation of testicular NO, nicotine decreased testicular NO significantly (*p* ≤ 0.001) compared to the normal group. One hundred and 150 mg/kg of both extracts produced a significant (*p* ≤ 0.001) increase in the concentration of NO, which equates to the effect of SC 5 mg/kg for the two extracts as compared to the negative control. Fifty milligrams per kilogram of both extracts did not cause any statistical change in the concentration of NO as compared to the negative control group.

The entire treatment did not have any effect on the GSH level, while the CAT activity was significantly decreased by nicotine when compared to the CAT activity of the normal control. From our results, treatment with aqueous extract showed a dose-independent significant increase (*p* ≤ 0.001) in CAT activity as compared to the negative control, just like the ethanol extract, which also had a dose-dependent increase of CAT activity as compared to the negative control. Treatment with Viagra had no significant difference in the activity of CAT after a comparison to the negative control. The *t*-test results showed that the two extracts had no statistical difference in their activity.

#### 3.2.2. Effect of Aqueous and Ethanol Extracts of *T. cistoides* on Seminal Vesicle Fructose, Testicular Protein, and Penile NO of Rats Exposed to Nicotine

Table 10 presents the effect of nicotine on the levels of total testicular proteins, seminal vesicle fructose concentration, and penile NO concentration and how ethanol and aqueous extracts affect these parameters. Nicotine significantly reduced the concentration of the total testicular proteins (*p* ≤ 0.001), seminal vesicle fructose (*p* ≤ 0.01), and penile NO (*p* ≤ 0.001) concentration after comparing with the normal control treated with distilled water. Treatment with the different doses of ethanol extract and aqueous extract increased the seminal vesicle fructose concentration significantly (*p* ≤ 0.001) in a dose-independent manner when compared to the negative control, unlike the extracts' effect on total testicular protein concentration, where both extracts increased the concentration in a dose-dependent manner, with the ethanol extract seen to have increased protein concentration significantly (*p* ≤ 0.001) more than the aqueous extract (*p* ≤ 0.01) following the treatment with 150 mg/kg as compared to the nicotine-treated animals. The effect of the extract on penile NO was dose dependent, with aqueous extract seen to have a greater effect (*p* ≤ 0.001) at 150 mg/kg of body weight than the same dose of ethanol extract when compared to the negative control. Treatment with SC did not increase the seminal vesicle fructose concentration and total testicular protein concentration caused by nicotine but was able to significantly increase penile NO concentration compared to the negative control group. The results of the *t*-test reveal no statistical difference in the performances of ethanol and aqueous extracts.

### 3.3. Effect of Aqueous and Ethanol Extracts of *T. cistoides* on Male Reproductive Hormones of Rats Exposed to Nicotine

After the assessment of the serum levels of testosterone, FSH, LH, and testicular levels of testosterone, the results were presented in Table 11. Exposure to nicotine significantly (*p* ≤ 0.001) reduced the serum and testicular testosterone levels and serum FSH and LH levels as compared to the normal control treated with distilled water. Following treatment with the plant extracts, the aqueous extract exhibited a dose-independent increase (*p* ≤ 0.001), unlike the ethanol extract, where all the doses significantly (*p* ≤ 0.001) increased the serum testosterone levels as compared to the negative control group. Fifty milligrams per kilogram of ethanol and aqueous extracts showed no significant increase in testicular testosterone concentration, while 100 and 150 mg/kg significantly (*p* ≤ 0.001) increased the testicular concentration of testosterone as compared to the negative control.

One hundred and 150 mg/kg of both extracts significantly (*p* ≤ 0.001) increased the serum concentration of FSH, while the ethanol extract at 50 mg/kg significantly increased FSH, unlike 50 mg/kg of the aqueous extract, which showed no statistical difference in FSH concentration when compared to the negative control treated with nicotine. LH assessment revealed that all doses of both extracts significantly increased the serum concentration (*p* ≤ 0.001) of LH compared to the negative control. According to the results of a *t*-test, the two extracts had similar effects.

### 3.4. Effect of *T. cistoides* Aqueous and Ethanol Extracts on the Histological Architecture of the Testis Exposed to Nicotine

**Figure 1:**
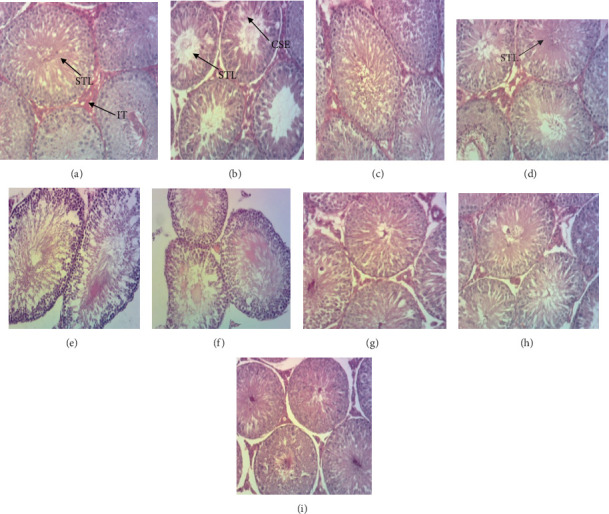
Microphotograph of the testis cross-sections showing the effect of *T. cistoides* on the histological structure of rat testis: (a) normal control, (b) negative control, (c) positive control, (d) 50 mg/kg aqueous extract, (e) 100 mg/kg aqueous extract, (f) 150 mg/kg aqueous extract, (g) 50 mg/kg ethanol extract, (h) 100 mg/kg ethanol extract, and (i) 150 mg/kg ethanol extract. STL = seminiferous tube lumen; IT = interstitial tissue; CSE = clarification of seminiferous epithelium (hematoxylin–eosin staining, 100×).

Figure 1 shows the cross-sections of the testes of experimental rats exposed to nicotine and treated with the aqueous and ethanol extract of *T. cistoides*. In a normal group of animals given distilled water, the seminiferous tubule lumen (STL) shows male sex cells (spermatogonia) at different stages of development from the periphery to the lumen (Figure 1a). We have also observed in the normal control group that there is a normal structure of interstitial tissue (IT) (Figure 1a). The nicotine-exposed group showed histopathological alterations of the testis marked by clarification of the seminiferous epithelium (CSE) and a decrease in sperm density in the seminiferous lumen (Figure 1b). The alterations seen in Figure 1b are being corrected by the different doses of ethanol and aqueous extracts, as seen in Figures 1c, 1d, 1e, 1f, 1g, 1h, and 1i.

## 4. Discussion

The use of medicinal plants for the treatment of animal and human diseases is as old as the existence of mankind given their safe and natural sources [31]. One of the approved areas of research in the fields of ethnopharmacology is the use of medicinal plants for the management of sexual dysfunction in men and women due to their global use in traditional medicine for the treatment of sexual disorders [32]. Because of the high cost and the side effects of existing synthetic drugs, the development of drugs from plants has formed the basis of in vivo research in the past decades, most especially in reproductive health due to their contributions to human sexual well-being [33]. Results from this work on *T. cistoides* showed similar trends with past research.

A decrease in ML and PEL is inversely proportional to sexual motivation, vigor, and desire according to the results of this study. These are signs of sexual arousal testifying to the aphrodisiac properties of the ethanol and aqueous extracts of *T. cistoides*. The decrease in ML and PEL and an increase in EL (delayed ejaculation for sexual satisfaction) are in conformity with the results of [32] who reported that the aqueous and hydroalcoholic extracts of the leaf and root bark of *Citropsis articulata* could reduce ML, EL, and PEL in male Wistar rats. The mechanism of action of plants with aphrodisiac properties is linked to various phytochemicals, like saponins, alkaloids, and flavonoids [32, 34]. Alkaloids, which are a component of medicinal plants, have been reported to increase penile erection and cause the dilation of blood vessels in the penis. Saponins are known to act on the central nervous system and gonadal tissues, leading to enhancement of libido and copulatory performance through facilitation of penile erection. Longer ejaculation latencies in the extract-treated groups are a sign that the animal, with the help of the aqueous and ethanol extract, is able to sustain an erection long enough for sexual satisfaction [31].

PLF, MF, IF, and EF are pivotal in the evaluation of sexual enhancement using the animal model. Our results on PLF, MF, IF, and EF are in agreement with the report of [35]. The authors of [35] proved that crude aqueous leaf extract of *Citropsis articulata and Mystroxylon aethiopicum* could increase MF and IF in male albino rats. Our results are also in synergy with that of [31] who evaluated the aphrodisiac potentials of the aqueous extract of *Hibiscus asper* leaves in male Wistar rats. Mount and intromission frequencies are useful biomarkers of vigor, libido, and potency [33]. Increase in MF, IF, and PLF in the extract-treated groups can be attributed to the increased sexual potency caused by the extracts as compared to the negative control group treated with nicotine. Increased sexual potency is reflected in the efficiency of erection, penile orientation, and the ease with which ejaculatory reflexes occur and hence the increase in the number of ejaculations in the extract-treated animals according to our results.

Proper functioning of the male reproductive system is androgen dependent; the assessment of testicular testosterone, serum testosterone, FSH, and LH gives a clear understanding of the mechanism through which plant extracts affect male reproductive potential [36]. According to our results, the plant extracts produced a significant effect on the hypothalamic–pituitary–gonadal (HPG) axis, demonstrated by a significant increase in the serum levels of LH and FSH, particularly in groups treated with 150 mg/kg of body weight of *T. cistoides* extracts. Testosterone plays a central role in the regulation of male sexual behavior and spermatogenesis. In this study, we measured testosterone levels both in the serum and testicular tissue to provide a more comprehensive understanding of the androgenic effects of *T. cistoides* extracts. Measurement of serum testosterone reflects the circulating levels of the hormone, which are indicative of systemic androgenic activity and endocrine function, particularly of the HPG axis. On the other hand, intratesticular testosterone levels are crucial for normal spermatogenesis and local testicular function. Intratesticular testosterone concentrations are typically much higher than serum levels and are maintained by Leydig cell activity within the testes. Assessing both compartments allows us to determine whether the extracts exert their effects primarily via systemic hormonal modulation or through direct action on testicular steroidogenesis [37]. In the context of nicotine-induced sexual dysfunction (which is known to impair both serum testosterone levels and Leydig cell function), evaluating both serum and testicular testosterone provides mechanistic insight into how *T. cistoides* mitigates these effects. This dual measurement strategy strengthens our findings by linking observed behavioral and reproductive changes with hormonal correlates at both systemic and local (testicular) levels [37]. The observed increase in both serum and testicular testosterone levels suggests that the extracts enhance androgen production not only by modulating systemic endocrine pathways but also by directly stimulating testicular steroidogenesis. This dual effect likely contributes to the improvement in sexual function observed in nicotine-treated rats. This is in line with the report of [35, 38, 39]. Nicotine is able to alter the production of FSH, LH, and testosterone, impairing spermatogenesis and male sexual functions. Our findings agree with the results of [11, 40]. An increase in LH, FSH, and testosterone are indicators of the action of the plant extracts on the hypothalamus, causing the release of gonadotropin-releasing hormone, which stimulates the release of LH and FSH. LH acts on Leydig cells, causing the production of testosterone in the testis, as seen in this study [41]. The increase in testosterone production induced by the plant extract justifies the aphrodisiac or sex-enhancing behavior observed in this study since testosterone is responsible for libido and promotes erection [41]. Normally, higher blood levels of testosterone exert negative feedback on the anterior pituitary level of gonadotropins (FSH and LH) [41]. In this study, we observed that the increased serum level of testosterone was accompanied by a sustained increase in serum levels of FSH and LH. This result can be explained by the fact that the plant extract may contain bioactive molecules able to cross the blood–brain barrier to stimulate the hypothalamus or the anterior pituitary, therefore bypassing the normal negative feedback of serum testosterone on the brain, promoting the effect of these hormones. A similar result was obtained by [39] who studied the aphrodisiac property of aqueous and methanolic extracts of *Raphia vinifera* (Arecaceae) in sexually experienced male rats.

Seminal vesicle–derived fructose serves as an androgen-dependent metabolic substrate for spermatozoa, with secretion declining in low-testosterone states and age [42]. While absolute fructose concentration inversely correlates with sperm count due to consumption postejaculation, “corrected fructose” more accurately reflects seminal vesicle secretory capacity and is reduced in men with low serum testosterone or obstructive pathology [43]. Recent findings also suggest seminal vesicle dysfunction (potentially indicated by low corrected fructose) may be associated with broader sexual dysfunctions such as erectile dysfunction, delayed or premature ejaculation, and diminished libido [42, 44]. Within the context of *T. cistoides*, which exhibits androgenic and aphrodisiac effects, preservation or enhancement of fructose secretion may reflect improved accessory gland function and, by extension, sexual function [42]. Future studies should measure corrected fructose alongside functional sexual outcomes to strengthen this mechanistic link. Testicular proteins are one of the constituents that ensure the maturation of spermatozoa, implying higher protein content, as seen in the extract-treated group, can be associated with improved fertility as a result of treatment with *T. cistoides* extracts [33]. Our results on testicular protein and vesicular fructose concentrations are in agreement with the report of [31] who reported an increase in total protein concentration in rats treated with aqueous extract of *Hibiscus asper* (Malvaceae) leaves in male Wistar rats and [43] who observed an increase in vesicular fructose concentration after treating male albino rats with extract of *Nymphaea lotus* at doses of 75 and 150 mg once daily for 55 days. The increase in testicular protein levels can be attributed to the androgenicity (testosterone), which enhances protein synthesis and sperm maturation [44]. Seminal vesicle fructose is known to energize spermatozoa. The significant increase in seminal vesicle fructose concentration in the extract-treated groups could be due to some bioactive substances such as flavonoids and terpenoids in the plant extracts [31]. Similarly, it could be triggered by the higher levels of testosterone, which is the main androgen responsible for development and proper functioning of male reproductive organs [40].

After experimenting the effect of the extracts on reproductive organs, we realized that our results were in synergy with the results of [32]. They investigated the in vivo aphrodisiac effect of the aqueous and hydroalcoholic extracts of the leaf and root bark of *Citropsis articulata* in male Wistar rats and discovered an insignificant increase in the reproductive weight of organs. The slight increase in the reproductive weight of organs in the extract-treated groups could be attributed to the anabolic effect due to increased testosterone secretion stimulated by the extract and their secretory effect as compared to that in the negative control group. The short duration of treatment (9 days) could be responsible for the insignificant increase in the weight of reproductive organs and that some organs like the penis at maturity might have attained maximum growth and may not respond to increased androgen levels. Our results on penile NO are in line with the reports of several authors [31, 40, 45, 46]. The increase in the penile NO, which is androgen dependent, justifies once more the aphrodisiac and androgenic effects of these plant extracts. In fact, during the process of erection, there is an activation of the parasympathetic nervous system supplying the penis, leading to the production of NO, which induces the vasorelaxation of penile arteries and subsequently the filling of blood in the erectile bodies [33, 47].

The testis, like other organs of the body, has an antioxidant system that ensures a balance between reactive oxygen species and antioxidants. The significant increase in the activity of testicular SOD, CAT, and NO and a decrease in TBARS concentration, as seen in this study, could be a demonstration of the plant extracts' efficiency in ensuring a balance between the antioxidant and free radicals in the testis induced by nicotine, as seen in the negative control. The protective effect of the plant extracts against nicotine toxicity on the testis may be due to the modulation of antioxidant systems through direct scavenging of reactive oxygen species and decreased lipid peroxidation induced by nicotine, as seen in the groups treated with 150 mg/kg of the plant extracts. This shows a significant improvement in the testicular antioxidant status [48]. SOD removes superoxide anions by converting them to hydrogen peroxide and thus diminishing the toxic effect caused by free radicals; CAT decomposes hydrogen peroxide and protects the tissues from highly reactive hydroxyl radicals.

Plants with aphrodisiac properties act generally through the NO-based mechanism and androgen-based mechanism. NO causes relaxation of the trabecular smooth muscles of the corpus cavernous leading to a decreased vascular resistance and increased blood flow to the penis resulting in an erection, while testosterone is converted to estradiol in the hypothalamus which increases sexual functions. Similarly, penile erections are also initiated through the cyclic adenosine monophosphate pathway (cAMP) via the mediation of corporal smooth muscles, respective enzymes, and proteins such as prostaglandin and the protein kinase G which causes smooth muscle relaxation and also increases the concentration of Ca^2+^. This induces a loss of the contractile tone of the penile smooth muscles and increases blood flow in the cavernous body thus yielding an erection [49]. The effectiveness of plants used as aphrodisiacs is believed to be through various mechanisms such as vasodilation, elevation of androgens, gonadotropin, and generation of NO [33]. *T. cistoides* extracts used in this study did not only exhibit aphrodisiac and androgenic properties as seen in sexual behavioral parameters and androgen-dependent parameters. The plant extracts also preserve the histological architectures of the testis from the damaging effect of nicotine as seen in the negative control. The histological images indicated a normal sperm density in the seminiferous lumen and the different stages of sperm development which is in line with studies of [32, 35].

SC, which is the reference drug for the treatment of erectile dysfunction, is a selective inhibitor of cGMP-specific PDE5 that facilitates penile erection through the relaxation of the corpus cavernosum, an event mediated by NO and cGMP [47]. Treatment with this drug enhanced the sexual well-being of male Wistar rats, which was demonstrated through the significant increase in MF, EF, IF, EL, and PLF and a significant decrease in the ML and PEL as compared to the negative control treated with nicotine. These results are similar to those of [31, 33]. Based on our results, Viagra had neither antioxidant characteristics nor androgenic effects, given that treatment with Viagra did not have any effect on the parameters of oxidative stress and androgen-dependent parameters. This suggests an advantage of our plant extracts over Viagra, which at the same time exhibited aphrodisiac, androgenic, and antioxidant effects.

In order to investigate the difference in the effect of the two extracts, a *t*-test was conducted to determine which of the extracts was more efficient in managing sexual dysfunctions induced by nicotine. Generally, no significant difference was recorded in the action of the ethanol and aqueous extracts after a comparison of the means of the various parameters evaluated for the respective doses of the extracts. The comparable androgenic and aphrodisiac effects observed with both the aqueous and ethanol extracts suggest that the bioactive constituents responsible for these activities may be present across different polarity ranges. This indicates that multiple phytochemical classes such as saponins, flavonoids, and possibly alkaloids or steroidal compounds may act synergistically or share similar mechanisms of action. The effectiveness of the aqueous extract also supports the traditional use of *T. cistoides* in decoctions or water-based preparations, while the ethanolic extract reinforces the pharmacological potential of more lipophilic constituents. These findings highlight the therapeutic versatility of the plant and suggest that both traditional and modern extraction methods may yield biologically active preparations with similar efficacy. These results are in agreement with that of [50], who did a fertility assessment of the male albino rats (Wistar strain) treated with aqueous and ethanol leaf extracts of *Euphorbia hirta* Linn and reported that both extracts had improved sperm characteristics after 14 days of treatment.

It is important to acknowledge that, although the study focused primarily on hormonal, biochemical, and behavioral markers of sexual function, not all facets of male sexual dysfunction were directly assessed. Parameters such as premature ejaculation, low libido, and anorgasmia were indirectly evaluated through behavioral observations. Notably, treatment with *T. cistoides* extracts delayed ejaculation and increased the number of ejaculations compared to the nicotine-treated group, suggesting a potential improvement in ejaculatory function and sexual motivation. However, further studies employing more specific behavioral assays are necessary to comprehensively assess these aspects. This limitation, along with others, highlights the need for broader evaluations in future investigations.

## 5. Conclusion

*T. cistoides* aqueous and ethanol extracts possess aphrodisiac properties, as proven from this study by the increase in MF, IF, PLF, EF, and EL compared to the negative control and reduction in the ML and PEL. The plant extracts also exhibited androgenic effects, evidenced by their stimulatory effects on the HPG axis by the increase in FSH and LH, testosterone in the serum, testicular testosterone, total testicular protein concentration, and seminal vesicle fructose concentration. *T*. *cistoides* extracts also showed antioxidant properties and have been found to increase the activity of testicular SOD, CAT, and the level of NO and GSH while decreasing the testicular level of TBARS. Similarly, the plant is thought to enhance erection through vasodilation, elevation of gonadotropins and androgens, and generation of penile NO, therefore supporting its use in traditional medicine as an aphrodisiac.

## Figures and Tables

**Table 1:** Effect of aqueous and ethanol extracts of *T. cistoides* on mount latency (in seconds) of male rats exposed to nicotine.

**Days**	**Extracts**	**Control groups**			**Extract-treated groups**			**F** ** value**	**p** ** value**
		**Normal control (distilled water 5 mL/kg of body weight)**	**Negative control (nicotine 1 mg/kg of body weight)**	**Positive control (sildenafil citrate 5 mg/kg of body weight)**	***T. cistoides* (50 mg/kg of body weight)**	***T. cistoides* (100 mg/kg of body weight)**	***T. cistoides* (150 mg/kg of body weight)**		
Day 1	Aqueous	10.40 ± 1.327^c^	34.00 ± 2.683	10.00 ± 0.707^c^	10.60 ± 0.678^c^	5.800 ± 0.3742^c^	4.600 ± 0.400^c^	*F*(5, 24) = 68.55	*p* ≤ 0.001
	Ethanol				9.800 ± 0.663^c^	5.600 ± 0.2449^c^	3.600 ± 0.511^c^	*F*(5, 24) = 71.22	*p* ≤ 0.001
	*t*-test/*p*				*t* = 0.84/0.426	*t* = 0.45/0.6666	*t* = 1.54/0.164		

Day 5	Aqueous	12.20 ± 0.663^c^	36.60 ± 2.04	6.600 ± 0.509^c^	10.80 ± 0.916^c^	4.200 ± 0.4899^c^	2.200 ± 0.374^c^	*F*(5, 24) = 156.4	*p* ≤ 0.001
	Ethanol				2.600 ± 0.509^c^	4.000 ± 0.4472^c^	3.400 ± 0.244^c^	*F*(5, 24) = 190.4	*p* ≤ 0.001
	*t*-test/*p*				*t* = 7.82/0.001	*t* = 0.30/0.7707	*t* = 2.68/0.0278		

Day 9	Aqueous	8.600 ± 0.511^c^	32.00 ± 1.58	6.200 ± 0.735^c^	7.400 ± 0.245^c^	3.000 ± 0.548^c^	1.400 ± 0.400^c^	*F*(5, 24) = 197.8	*p* ≤ 0.001
	Ethanol				8.200 ± 0.374^c^	3.600 ± 0.748^c^	1.800 ± 0.374^c^	*F*(5, 24) = 177.3	*p* ≤ 0.001
	*t*-test/*p*				*t* = 1.79/0.1114	*t* = 0.65/0.5358	*t* = 0.73/0.4860		

*Note:* Values in the table represent mean ± SEM of five rats per group, while superscript letter “c” represents significance with *p* ≤ 0.001 when the normal control group treated with distilled water and the plant extract–treated groups are compared to the negative control treated with nicotine.

**Table 2:** Results of the effect of aqueous and ethanol extract of *T. cistoides* on mount frequency of male rats exposed to nicotine.

	**Extracts**	**Control groups**			**Extract-treated groups**			**F** ** value**	**p** ** value**
		**Normal control (distilled water 5 mL/kg of body weight)**	**Negative control (nicotine 1 mg/kg of body weight)**	**Positive control (sildenafil citrate 5 mg/kg of body weight)**	***T. cistoides* (50 mg/kg of body weight)**	***T. cistoides* (100 mg/kg of body weight)**	***T. cistoides* (150 mg/kg of body weight)**		
Day 1	Aqueous	21.80 ± 2.634^ns^	17.60 ± 1.503	21.20 ± 2.518^ns^	23.00 ± 2.429^ns^	24.60 ± 3.868^ns^	26.00 ± 4.195^ns^	*F*(5, 24) = 0.955	*p* ≥ 0.05
	Ethanol				19.60 ± 3.156^ns^	23.80 ± 1.772^ns^	31.40 ± 2.731^b^	*F*(5, 24) = 3.838	*p* ≤ 0.05
	*t*-test/*p*				*t* = 0.85/0.4181	*t* = 0.19/0.8555	*t* = 1.08/0.3122		

Day 5	Aqueous	26.80 ± 1.356^b^	15.00 ± 1.342	25.40 ± 1.990^b^	27.80 ± 3.121^c^	28.00 ± 0.8367^c^	28.20 ± 1.828^c^	*F*(5, 24) = 7.310	*p* ≤ 0.001
	Ethanol				27.00 ± 1.975^b^	27.40 ± 1.887^b^	30.80 ± 2.396^c^	*F*(5, 24) = 8.003	*p* ≤ 0.001
	*t*-test/*p*				*t* = 0.22/0.8339	*t* = 0.29/0.7787	*t* = 0.86/0.413		

Day 9	Aqueous	28.20 ± 1.393^b^	16.80 ± 0.5831	27.20 ± 1.855^b^	30.20 ± 1.828^c^	31.20 ± 2.800^c^	36.80 ± 1.655^c^	*F*(5, 24) = 13.30	*p* ≤ 0.001
	Ethanol				28.20 ± 0.8602^b^	29.80 ± 1.562^b^	35.60 ± 4.320^c^	*F*(5, 24) = 8.450	*p* ≤ 0.001
	*t*-test/*p*				*t* = 0.99/0.3511	*t* = 0.45/0.6739	*t* = 0.26/0.802		

*Note:* Values in the table represent mean ± SEM of five rats per group. Superscript letter “ns” represents no significant difference with *p* ≥ 0.05. Superscript letter “b” represents significance with *p* ≤ 0.01. Superscript letter “c” represents significance with *p* ≤ 0.001 when the normal control group treated with distilled water and the plant extract–treated groups are compared to the negative control treated with nicotine.

**Table 3:** Result of the effect of aqueous and ethanol extracts of *T. cistoides* on ejaculatory latency (in seconds) of male rats exposed to nicotine.

**Days**	**Extracts**	**Control groups**			**Extract-treated groups**			**F** ** value**	**p** ** value**
		**Normal control (distilled water 5 mL/kg of body weight)**	**Negative control (nicotine 1 mg/kg of body weight)**	**Positive control (sildenafil citrate 5 mg/kg of body weight)**	***T. cistoides* (50 mg/kg of body weight)**	***T. cistoides* (100 mg/kg of body weight)**	***T. cistoides* (150 mg/kg of body weight)**		
Day 1	Aqueous	365.0 ± 16.12^c^	182.0 ± 12.47	362.8 ± 13.15^c^	311.6 ± 24.86^b^	373.4 ± 22.82^c^	401.0 ± 41.09^c^	*F*(5, 24) = 11.04	*p* ≤ 0.001
	Ethanol				323.8 ± 6.492^c^	343.6 ± 14.19^c^	355.0 ± 13.67^c^	*F*(5, 24) = 29.07	*p* ≤ 0.001
	*t*-test/*p*				*t* = 0.47/0.6477	*t* = 1.11/0.2996	*t* = 1.06/0.3191		

Day 5	Aqueous	311.2 ± 28.73^a^	200.6 ± 14.73	310.2 ± 28.97^a^	246.6 ± 10.30^ns^	398.0 ± 24.02^c^	557.0 ± 18.54^c^	*F*(5, 24) = 33.13	*p* ≤ 0.001
	Ethanol				310.6 ± 19.87^b^	399.2 ± 33.22^c^	409.8 ± 24.44^c^	*F*(5, 24) = 8.683	*p* ≤ 0.001
	*t*-test/*p*				*t* = 2.86/0.0212	*t* = 0.03/0.9774	*t* = 4.81/0.0014		

Day 9	Aqueous	319.8 ± 4.521^c^	214.6 ± 7.935	267.0 ± 11.85^ns^	314.4 ± 12.89^c^	396.2 ± 9.484^c^	421.8 ± 20.64^c^	*F*(5, 24) = 39.86	*p* ≤ 0.001
	Ethanol				283.2 ± 12.10^c^	397.2 ± 15.47^c^	514.8 ± 30.77^c^	*F*(5, 24) = 44.87	*p* ≤ 0.001
	*t*-test/*p*				*t* = 1.77/0.1156	*t* = 0.06/0.9574	*t* = 2.51/0.0364		

*Note:* Values represent mean ± SEM of five rats per group. Superscript letter “ns” represents no significant difference with *p* ≥ 0.05. Superscript letter “a” represents significance with *p* ≥ 0.05. Superscript letter “b” represents significance with *p* ≤ 0.01. Superscript letter “c” represents significance with *p* ≤ 0.001 when the normal control group treated with distilled water and the plant extract–treated groups are compared to the negative control treated with nicotine.

**Table 4:** Results of the effect of aqueous and ethanol extracts of *T. cistoides* on ejaculatory frequency of male rats exposed to nicotine.

**Days**	**Extracts**	**Control groups**			**Extract-treated groups**			**F** ** value**	**p** ** value**
		**Normal control (distill water 5 mg/kg of body weight)**	**Negative control (nicotine 1 mg/kg of body weight)**	**Positive control (sildenafil citrate 5 mg/kg of body weight)**	**Dose 1 of *T. cistoides* (50 mg/kg of body weight)**	**Dose 2 of *T. cistoides* (100 mg/kg of body weight)**	**Dose 3 of *T. cistoides* (150 mg/kg of body weight)**		
Day 1	Aqueous	1.600 ± 0.244^ns^	1.400 ± 0.245	1.00 ± 0.00^ns^	1.600 ± 0.245^ns^	1.600 ± 0.245^ns^	1.800 ± 0.200^ns^	*F*(5, 24) = 1.600	*p* ≥ 0.05
	Ethanol				1.600 ± 0.245^ns^	1.800 ± 0.200^ns^	1.800 ± 0.200^ns^	*F*(5, 24) = 2.000	*p* ≥ 0.05
	*t*-test/*p*				*t* = 0.00/0.999	*t* = 0.00/0.999	*t* = 0.000/0.0999		

Day 5	Aqueous	1.600 ± 0.245^a^	1.000 ± 0.000	2.200 ± 0.20^b^	1.600 ± 0.245^ns^	1.800 ± 0.200^ns^	2.200 ± 0.20^b^	*F*(5, 24) = 4.457	*p* ≤ 0.05
	Ethanol				1.600 ± 0.245^ns^	2.200 ± 0.200^b^	2.400 ± 0.2449^c^	*F*(5, 24) = 7.169	*p* ≤ 0.001
	*t*-test/*p*				*t* = 0.00/0.9999	*t* = 1.414/0.1950	*t* = 0.6325/0.5447		

Day 9	Aqueous	2.200 ± 0.2000^b^	1.00 ± 0.000	2.400 ± 0.245^c^	2.000 ± 0.000^b^	2.000 ± 0.316^b^	2.400 ± 0.2449^c^	*F*(5, 24) = 8.360	*p* ≤ 0.001
	Ethanol				2.000 ± 0.000^b^	2.200 ± 0.163^c^	2.200 ± 0.163^c^	*F*(5, 24) = 8.533	*p* ≤ 0.001
	*t*-test/*p*				*t* = 1.633/0.1411	*t* = 0.000/0.9999	*t* = 0.6325/0.5447		

*Note:* Values represent mean ± SEM of five rats per group. Superscript letter “ns” represents no significant difference with *p* ≥ 0.05. Superscript letter “a” represents significance with *p* ≤ 0.05. Superscript letter “b” represents significance with *p* ≤ 0.01. Superscript letter “c” represents significance with *p* ≤ 0.001 when the normal control group treated with distilled water and the plant extract–treated groups are compared to the negative control treated with nicotine.

**Table 5:** Results of the effect of aqueous and ethanol extracts of *T. cistoides* on postejaculatory interval (in seconds) of male rats exposed to nicotine.

**Days**	**Extracts**	**Control groups**			**Extract-treated groups**			**F** ** value**	**p** ** value**
		**Normal control (distill water 5 mg/kg of body weight)**	**Negative control (nicotine 1 mg/kg of body weight)**	**Positive control (sildenafil citrate 5 mg/kg of body weight)**	**Dose 1 of *T. cistoides* (50 mg/kg of body weight)**	**Dose 2 of *T. cistoides* (100 mg/kg of body weight)**	**Dose 3 of *T. cistoides* (150 mg/kg of body weight)**		
Day 1	Aqueous	360.2 ± 43.25^ns^	308.0 ± 19.20	352.6 ± 15.70^ns^	323.4 ± 14.77^ns^	313.2 ± 23.27^ns^	268.2 ± 60.48^ns^	*F*(5, 24) = 0.9663	*p* ≥ 0.05
	Ethanol				324.4 ± 38.85^ns^	293.2 ± 34.43^ns^	285.4 ± 25.36^ns^	*F*(5, 24) = 0.9798	*p* ≥ 0.05
	*t*-test/*p*				*t* = 0.02/0.9814	*t* = 0.48/0.6432	*t* = 0.26/0.7997		

Day 5	Aqueous	300.8 ± 18.55^a^	450.2 ± 18.37	352.6 ± 15.70^ns^	313.8 ± 14.55^a^	306.0 ± 24.00^a^	268.2 ± 60.48^b^	*F*(5, 24) = 4.566	*p* ≤ 0.01
	Ethanol				316.6 ± 39.45^a^	279.8 ± 33.24^a^	285.4 ± 25.36^a^	*F*(5, 24) = 3.166	*p* ≤ 0.05
	*t*-test/*p*				*t* = 0.07/0.9485	*t* = 0.64/0.5407	*t* = 0.26/0.7997		

Day 9	Aqueous	319.0 ± 17.84^b^	460.0 ± 14.14	351.2 ± 28.00^a^	358.8 ± 28.50^a^	296.4 ± 10.63^c^	231.4 ± 48.47^c^	*F*(5, 30) = 11.13	*p* ≤ 0.001
	Ethanol				347.0 ± 14.98^b^	260.8 ± 6.873^c^	281.6 ± 13.91^c^	*F*(5, 24) = 15.82	*p* ≤ 0.001
	*t*-test/*p*				*t* = 0.37/0.7235	*t* = 2.81/0.0227	*t* = 1.1/0.3487		

*Note:* Values represent mean ± SEM of five rats per group. Superscript letter “ns” represents no significant difference with *p* ≥ 0.05. Superscript letter “a” represents significance with *p* ≤ 0.05. Superscript letter “b” represents significance with *p* ≤ 0.01. Superscript letter “c” represents significance with *p* ≤ 0.001 when the normal control group treated with distilled water and the plant extract–treated groups are compared to the negative control treated with nicotine.

**Table 6:** Results of the effect of aqueous and ethanol extracts of *T. cistoides* on penile leaking frequency of male rats exposed to nicotine.

**Days**	**Extracts**	**Control groups**			**Extract-treated groups**			**F** ** value**	**p** ** value**
		**Normal control (distilled water 5 mg/kg of body weight)**	**Negative control (nicotine 1 mg/kg of body weight)**	**Positive control (sildenafil citrate 5 mg/kg of body weight)**	***T. cistoides* (50 mg/kg of body weight)**	***T. cistoides* (100 mg/kg of body weight)**	***T. cistoides* (150 mg/kg of body weight)**		
Day 1	Aqueous	21.80 ± 0.491^a^	14.60 ± 0.511	23.60 ± 2.112^c^	20.40 ± 0.812^ns^	22.20 ± 0.860^b^	25.40 ± 2.482^c^	*F*(5, 24) = 6.599	*p* ≤ 0.001
	Ethanol				21.60 ± 0.9274^a^	23.80 ± 1.772^c^	26.60 ± 2.315^c^	*F*(5, 24) = 6.865	*p* ≤ 0.001
	*t*-test/*p*				*t* = 0.97/0.3589	*t* = 0.81/0.4401	*t* = 0.35/0.7328		

Day 5	Aqueous	23.40 ± 1.965^a^	14.00 ± 1.049	27.60 ± 0.9798^c^	26.80 ± 2.596^b^	33.60 ± 1.470^c^	40.40 ± 2.731^c^	*F*(5, 24) = 21.71	*p* ≤ 0.001
	Ethanol				23.00 ± 0.8367^c^	29.40 ± 0.927^c^	37.60 ± 1.965^c^	*F*(5, 24) = 32.62	*p* ≤ 0.001
	*t*-test/*p*				*t* = 1.39/0.2011	*t* = 2.42/0.0421	*t* = 0.83/0.4294		

Day 9	Aqueous	23.00 ± 1.095^c^	11.20 ± 0.7348	24.60 ± 1.288^c^	24.40 ± 2.293^b^	36.20 ± 1.655^c^	39.40 ± 1.030^c^	*F*(5, 24) = 49.58	*p* ≤ 0.001
	Ethanol				21.00 ± 1.483^c^	33.40 ± 1.208^c^	44.00 ± 0.633^c^	*F*(5, 24) = 101.9	*p* ≤ 0.001
	*t*-test/*p*				*t* = 1.25/0.2484	*t* = 1.37/0.209	*t* = 3.81/0.0052		

*Note:* Values represent mean ± SEM of five rats per group. Superscript letter “ns” represents no significant difference with *p* ≥ 0.05. Superscript letter “a” represents significance with *p* ≤ 0.05. Superscript letter “b” represents significance with *p* ≤ 0.01. Superscript letter “c” represents significance with *p* ≤ 0.001 when the normal control group treated with distilled water and the plant extract–treated groups are compared to the negative control treated with nicotine.

**Table 7:** Results of the effect of aqueous and ethanol extracts of *T. cistoides* on intromission frequency of male rats exposed to nicotine.

**Days**	**Extracts**	**Control groups**			**Extract-treated groups**			**F** ** value**	**p** ** value**
		**Normal control (distilled water 5 mg/kg of body weight)**	**Negative control (nicotine 1 mg/kg of body weight)**	**Positive control (sildenafil citrate 5 mg/kg of body weight)**	***T. cistoides* (50 mg/kg of body weight)**	***T. cistoides* (100 mg/kg of body weight)**	***T. cistoides* (150 mg/kg of body weight)**		
Day 1	Aqueous	26.00 ± 0.7071^c^	15.20 ± 0.20	45.60 ± 1.503^c^	24.20 ± 1.594^c^	30.20 ± 1.158^c^	39.40 ± 1.166^c^	*F*(5, 24) = 89.61	*p* ≤ 0.001
	Ethanol				23.20 ± 1.655^c^	31.20 ± 0.800^c^	46.40 ± 0.871^c^	*F*(5, 24) = 135.8	*p* ≤ 0.001
	*t*-test/*p*				*t* = 0.44/0.675	*t* = 0.71/0.498	*t* = 4.81/0.001		

Day 5	Aqueous	20.20 ± 1.960^a^	14.40 ± 1.435	43.40 ± 1.166^c^	22.80 ± 1.020^c^	38.00 ± 1.304^c^	44.40 ± 0.400^c^	*F*(5, 24) = 99.27	*p* ≤ 0.001
	Ethanol				22.40 ± 0.678^c^	34.00 ± 1.517^c^	46.00 ± 0.894^c^	*F*(5, 24) = 94.12	*p* ≤ 0.001
	*t*-test/*p*				*t* = 0.33/0.752	*t* = 2.00/0.081	*t* = 1.63/0.141		

Day 9	Aqueous	21.20 ± 1.772^b^	12.80 ± 0.581	44.60 ± 1.288^c^	23.40 ± 1.536^c^	34.40 ± 1.631^c^	42.40 ± 1.288^c^	*F*(5, 24) = 81.51	*p* ≤ 0.001
	Ethanol				22.20 ± 034^c^	36.40 ± 1.166^c^	43.60 ± 1.631^c^	*F*(5, 24) = 108.0	*p* ≤ 0.001
	*t*-test/*p*				*t* = 0.71/0.501	*t* = 1.91/0.348	*t* = 0.58/0.581		

*Note:* Values represent mean ± SEM of five rats per group. Superscript letter “a” represents significance with *p* ≤ 0.05. Superscript letter “b” represents significance with *p* ≤ 0.01. Superscript letter “c” represents significance with *p* ≤ 0.001 when the normal control group treated with distilled water and the plant extract–treated groups are compared to the negative control treated with nicotine.

**Table 8:** Results of the effect of *T. cistoides* extracts on reproductive weight organs (grams) of rats exposed to nicotine.

**Organs**	**Extracts**	**Control groups**			**Extract-treated groups**			**F** ** value**	**p** ** value**
		**Normal control (distill water 5 mg/kg of body weight)**	**Negative control (nicotine 1 mg/kg of body weight)**	**Positive control (sildenafil citrate 5 mg/kg of body weight)**	**Dose 1 of *T. cistoides* (50 mg/kg of body weight)**	**Dose 2 of *T. cistoides* (100 mg/kg of body weight)**	**Dose 3 of *T. cistoides* (150 mg/kg of body weight)**		
Epididymis	Aqueous	0.525 ± 0.033^ns^	0.471 ± 0.015	0.459 ± 0.0206^ns^	0.523 ± 0.019^ns^	0.489 ± 0.021^ns^	0.483 ± 0.0216^ns^	*F*(5, 24) = 1.460	*p* ≥ 0.05
	Ethanol				0.451 ± 0.034^ns^	0.477 ± 0.008^ns^	0.544 ± 0.034^ns^	*F*(5, 24) = 2.090	*p* ≥ 0.05
	*t*-test/*p*				*t* = 0.16/0.8769	*t* = 4.92/0.0012	*t* = 0.24/0.8201		

Testis	Aqueous	1.438 ± 0.040^ns^	1.469 ± 0.084	1.465 ± 0.03^ns^	1.398 ± 0.031^ns^	1.530 ± 0.038^ns^	1.577 ± 0.045^ns^	*F*(5, 24) = 1.131	*p* ≥ 0.05
	Ethanol				1.454 ± 0.058^ns^	1.505 ± 0.043^ns^	1.598 ± 0.055^ns^	*F*(5, 24) = 3.674	*p* ≥ 0.05
	*t*-test/*p*				*t* = 1.35/0.2132	*t* = 2.59/0.0322	*t* = 0.88/0.4024		

Seminal vesicle	Aqueous	0.356 ± 0.016^ns^	0.332 ± 0.017	0.334 ± 0.031^ns^	0.382 ± 0.045^ns^	0.418 ± 0.008^ns^	0.404 ± 0.023^ns^	*F*(5, 24) = 1.894	*p* ≥ 0.05
	Ethanol				0.378 ± 0.032^ns^	0.373 ± 0.033^ns^	0.393 ± 0.061^ns^	*F*(5, 24) = 0.5010	*p* ≥ 0.05
	*t*-test/*p*				*t* = 0.09/0.9324	*t* = 1.34/0.2182	*t* = 0.16/0.8739		

Prostate	Aqueous	0.147 ± 0.018^ns^	0.149 ± 0.016	0.141 ± 0.018^ns^	0.152 ± 0.0141^ns^	0.183 ± 0.014^ns^	0.201 ± 0.031^ns^	*F*(5, 24) = 1.521	*p* ≥ 0.05
	Ethanol				0.141 ± 0.020^ns^	0.141 ± 0.015^ns^	0.159 ± 0.015^ns^	*F*(5, 24) = 1.685	*p* ≥ 0.05
	*t*-test/*p*				*t* = 0.39/0.7759	*t* = 2.08/0.0716	*t* = 0.55/0.5990		

Penis	Aqueous	0.175 ± 0.021	0.143 ± 0.014	0.141 ± 0.004^ns^	0.181 ± 0.007^ns^	0.181 ± 0.005^ns^	0.175 ± 0.005^ns^	*F*(5, 24) = 2.730	*p* ≥ 0.05
	Ethanol				0.181 ± 0.007^ns^	0.181 ± 0.005^ns^	0.175 ± 0.005^ns^	*F*(5, 24) = 2.730	*p* ≤ 0.05
	*t*-test/*p*				*t* = 1.03/0.3323	*t* = 1.93/0.0897	*t* = 0.53/0.6129		

*Note:* Values represent mean ± SEM of five rats per group. Superscript letter “ns” represents no significant difference with *p* ≥ 0.05 when the normal control group treated with distilled water and the plant extract–treated groups are compared to the negative control treated with nicotine.

**Table 9:** Results from the evaluation of the effect of the aqueous and ethanol extracts of *T. cistoides* on oxidative stress biomarkers in the testis of male rats exposed to nicotine.

**Parameters**	**Extracts**	**Control groups**			**Extract-treated groups**			**F** ** value**	**p** ** value**
		**Normal control (distill water 5 mg/kg of body weight)**	**Negative control (nicotine 1 mg/kg of body weight)**	**Positive control (sildenafil citrate 5 mg/kg of body weight)**	**Dose 1 of *T. cistoides* (50 mg/kg of body weight)**	**Dose 2 of *T. cistoides* (100 mg/kg of body weight)**	**Dose 3 of *T. cistoides* (150 mg/kg of body weight)**		
SOD (%I)	Aqueous	25.22 ± 1.096^c^	16.43 ± 1.181	18.98 ± 1.552^ns^	24.20 ± 0.806^c^	27.01 ± 1.114^c^	27.64 ± 0.771^c^	*F*(5, 24) = 16.49	*p* ≤ 0.001
	Ethanol				24.97 ± 2.247^a^	25.22 ± 2.137^a^	28.54 ± 1.499^c^	*F*(5, 24) = 7.353	*p* ≤ 0.001
	*t*-test/*p*				*t* = 0.32/0.7570	*t* = 0.74/0.4805	*t* = 0.53/0.6111		

TBARS (nmole/mg of proteins)	Aqueous	0.968 ± 0.089^a^	1.472 ± 0.065	1.328 ± 0.111^ns^	0.965 ± 0.122^a^	0.935 ± 0.123^b^	0.845 ± 0.06^b^	*F*(5, 24) = 6.570	*p* ≤ 0.001
	Ethanol				1.053 ± 0.065^ns^	0.891 ± 0.272^a^	0.847 ± 0.042^a^	*F*(5, 24) = 3.674	*p* ≤ 0.05
	*t*-test/*p*				*t* = 0.63/0.5469	*t* = 0.16/0.8777	*t* = 0.04/0.9729		

Testicular NO (*μ*mole/mL)	Aqueous	43.05 ± 0.681^c^	35.00 ± 0.178	44.66 ± 1.281^c^	36.92 ± 0.190^ns^	41.77 ± 0.9493^c^	41.29 ± 0.630^c^	*F*(5, 24) = 23.87	*p* ≤ 0.001
	Ethanol				36.62 ± 0.469^ns^	42.52 ± 0.762^c^	43.14 ± 1.412^c^	*F*(5, 24) = 19.33	*p* ≤ 0.001
	*t*-test/*p*				*t* = 0.59/0.5687	*t* = 0.61/0.5591	*t* = 1.21/0.2660		

GSH (*μ*M of protein)	Aqueous	1.188 ± 0.065^ns^	1.263 ± 0.14	1.424 ± 0.358^ns^	1.193 ± 0.088^ns^	1.278 ± 0.154^ns^	1.279 ± 0.122^ns^	*F*(5, 24) = 0.2195	*p* ≥ 0.05
	Ethanol				1.126 ± 0.012^ns^	1.076 ± 0.059^ns^	1.302 ± 0.035^ns^	*F*(5, 24) = 0.6064	*p* ≥ 0.05
	*t*-test/*p*				*t* = 0.75/0.4725	*t* = 1.23/0.2534	*t* = 0.18/0.8643		

CAT (*μ*mole of H_2_O_2_)	Ethanol	4.423 ± 0.296^c^	0.771 ± 0.191	1.778 ± 0.467^ns^	3.970 ± 0.125^c^	4.025 ± 0.115^c^	4.232 ± 0.492^c^	*F*(5, 24) = 23.02	*p* ≤ 0.001
	Aqueous				2.811 ± 0.471^b^	3.783 ± 0.338^c^	4.531 ± 0.367^c^	*F*(5, 24) = 14.09	*p* ≤ 0.001
	*t*-test/*p*				*t* = 2.38/0.0448	*t* = 0.89/0.3963	*t* = 1.18/0.2736		

*Note:* Values represent mean ± SEM of five rats per group. Superscript letter “ns” represents no significant difference with *p* ≤ 0.05. Superscript letter “a” represents significance with *p* ≤ 0.05. Superscript letter “b” represents significance with *p* ≤ 0.01. Superscript letter “c” represents significance with *p* ≤ 0.001 when the normal control group treated with distilled water and the plant extract–treated groups are compared to the negative control treated with nicotine.

**Table 10:** Results of the effect of the aqueous and ethanol extracts of *T. cistoides* on seminal vesicle fructose and testicular protein content of male rats exposed to nicotine.

**Parameters**	**Extracts**	**Control groups**			**Extract-treated groups**			**F** ** value**	**p** ** value**
		**Normal control (distill water 5 mg/kg of body weight)**	**Negative control (nicotine 1 mg/kg of body weight)**	**Positive control (sildenafil citrate 5 mg/kg of body weight)**	**Dose 1 of *T. cistoides* (50 mg/kg of body weight)**	**Dose 2 of *T. cistoides* (100 mg/kg of body weight)**	**Dose 3 of *T. cistoides* (150 mg/kg of body weight)**		
Seminal vesicle fructose (mM)	Aqueous	1.816 ± 0.073^b^	0.802 ± 0.031	1.014 ± 0.045^ns^	2.860 ± 0.324^c^	3.285 ± 0.157^c^	3.498 ± 0.145^c^	*F*(5, 30) = 51.48	*p* ≤ 0.001
	Ethanol				2.251 ± 0.365^c^	3.333 ± 0.061^c^	3.556 ± 0.192^c^	*F*(5, 24) = 42.62	*p* ≤ 0.001
	*t*-test/*p*				*t* = 1.13/0.293	*t* = 0.24/0.810	*t* = 0.22/0.831		

Protein (mg/g)	Aqueous	0.301 ± 0.014^c^	0.145 ± 0.013	0.157 ± 0.017^ns^	0.249 ± 0.021^a^	0.265 ± 0.034^a^	0.278 ± 0.029^b^	*F*(5, 24) = 8.346	*p* ≤ 0.001
	Ethanol				0.281 ± 0.039^b^	0.306 ± 0.028^c^	0.337 ± 0.018^c^	*F*(5, 24) = 12.48	*p* ≤ 0.001
	*t*-test/*p*				*t* = 0.70/0.501	*t* = 1.77/0.111	*t* = 0.74/0.476		

Penile NO (*μ*mole/mL)	Aqueous	40.14 ± 0.220^c^	30.17 ± 0.583	38.31 ± 1.045^c^	35.41 ± 0.383^c^	36.00 ± 0.528^c^	37.71 ± 0.978^c^	*F*(5, 24) = 24.82	*p* ≤ 0.001
	Ethanol				35.52 ± 0.176^b^	35.32 ± 1.631^b^	37.71 ± 1.13^c^	*F*(5, 24) = 13.17	
	*t*-test/*p*				*t* = 0.26/0.805	*t* = 0.41/0.703	*t* = 1.34/0.999		

*Note:* Values represent mean ± SEM of five rats per group. Superscript letter “ns” represents no significant difference with *p* ≥ 0.05. Superscript letter “a” represents significance with *p* ≤ 0.05. Superscript letter “b” represents significance with *p* ≤ 0.01. Superscript letter “c” represents significance with *p* ≤ 0.001 when the normal control group treated with distilled water and the plant extract–treated groups are compared to the negative control treated with nicotine.

**Table 11:** Results from the evaluation of the effect of aqueous and ethanol extracts of *T. cistoides* on the levels of testosterone, follicle-stimulating hormone, and luteinizing hormone.

**Parameters**	**Extracts**	**Control groups**			**Extract-treated groups**			**F** ** value**	**p** ** value**
		**Normal control (distill water 5 mg/kg of body weight)**	**Negative control (nicotine 1 mg/kg of body weight)**	**Positive control (sildenafil citrate 5 mg/kg of body weight)**	**Dose 1 of *T. cistoides* (50 mg/kg of body weight)**	**Dose 2 of *T. cistoides* (100 mg/kg of body weight)**	**Dose 3 of *T. cistoides* (150 mg/kg of body weight)**		
Serum testosterone (ng/mL)	Aqueous	1.606 ± 0.053^c^	0.079 ± 0.006	0.366 ± 0.034^ns^	0.40 ± 0.054^ns^	0.599 ± 0.054^b^	1.002 ± 0.165^c^	*F*(5, 24) = 48.68	*p* ≤ 0.001
	Ethanol				0.432 ± 0.024^c^	0.487 ± 0.051^c^	0.865 ± 0.041^c^	*F*(5, 24) = 198.1	*p* ≤ 0.001
	*t*-test/*p*				*t* = 0.537/0.6059	*t* = 1.532/0.1640	*t* = 0.804/0.4447		

Testicular testosterone (ng/mL)	Aqueous	40.77 ± 2.576^c^	20.01 ± 1.481	24.51 ± 0.694^ns^	27.11 ± 1.499^ns^	40.57 ± 1.826^c^	48.21 ± 3.178^c^	*F*(5, 24) = 29.90	*p* ≤ 0.001
	Ethanol				24.91 ± 0.732^ns^	34.36 ± 1.911^c^	38.93 ± 1.698^c^	*F*(5, 24) = 26.99	*p* ≤ 0.001
	*t*-test/*p*				*t* = 1.318/0.2240	*t* = 2.351/0.0466	*t* = 2.575/0.0329		

Serum FSH (mIU/mL)	Aqueous	0.959 ± 0.041^c^	0.402 ± 0.035	0.644 ± 0.049^ns^	0.472 ± 0.041^ns^	0.943 ± 0.053^c^	1.108 ± 0.112^c^	*F*(5, 24) = 22.47	*p* ≤ 0.001
	Ethanol				0.709 ± 0.021^b^	0.970 ± 0.046^c^	0.973 ± 0.073^c^	*F*(5, 24) = 24.61	*p* ≤ 0.001
	*t*-test/*p*				*t* = 5.170/0.0009	*t* = 0.375/0.7171	*t* = 1.005/0.3442		

Serum LH (mIU/mL)	Aqueous	10.17 ± 0.370^c^	1.88 ± 0.293	3.64 ± 0.451^ns^	6.74 ± 0.757^c^	8.62 ± 0.386^c^	10.58 ± 0.751^c^	*F*(5, 24) = 44.27	*p* ≤ 0.001
	Ethanol				8.01 ± 0.627^c^	10.34 ± 0.673^c^	11.15 ± 0.282^c^	*F*(5, 24) = 66.92	*p* ≤ 0.001
	*t*-test/*p*				*t* = 1.295/0.2315	*t* = 2.214/0.0578	*t* = 0.717/0.494		

*Note:* Values represent mean ± SEM of five rats per group. Superscript letter “ns” represents no significant difference with *p* ≥ 0.05. Superscript letter “a” represents significance with *p* ≤ 0.05. Superscript letter “b” represents significance with *p* ≤ 0.01. Superscript letter “c” represents significance with *p* ≤ 0.001 when the normal control group treated with distilled water and the plant extract–treated groups are compared to the negative control treated with nicotine.

## Data Availability

Data are available on request due to privacy/ethical restrictions.
